# Analysis of the Fibroblast Growth Factor System Reveals Alterations in a Mouse Model of Spinal Muscular Atrophy

**DOI:** 10.1371/journal.pone.0031202

**Published:** 2012-02-13

**Authors:** Niko Hensel, Andreas Ratzka, Hella Brinkmann, Lars Klimaschewski, Claudia Grothe, Peter Claus

**Affiliations:** 1 Institute of Neuroanatomy, Hannover Medical School, Hannover, Germany; 2 Center for Systems Neuroscience, Hannover, Germany; 3 Division of Neuroanatomy, Innsbruck Medical University, Innsbruck, Austria; Consejo Superior de Investigaciones Cientificas, Spain

## Abstract

The monogenetic disease Spinal Muscular Atrophy (SMA) is characterized by a progressive loss of motoneurons leading to muscle weakness and atrophy due to severe reduction of the Survival of Motoneuron (SMN) protein. Several models of SMA show deficits in neurite outgrowth and maintenance of neuromuscular junction (NMJ) structure. Survival of motoneurons, axonal outgrowth and formation of NMJ is controlled by neurotrophic factors such as the Fibroblast Growth Factor (FGF) system. Besides their classical role as extracellular ligands, some FGFs exert also intracellular functions controlling neuronal differentiation. We have previously shown that intracellular FGF-2 binds to SMN and regulates the number of a subtype of nuclear bodies which are reduced in SMA patients. In the light of these findings, we systematically analyzed the FGF-system comprising five canonical receptors and 22 ligands in a severe mouse model of SMA. In this study, we demonstrate widespread alterations of the FGF-system in both muscle and spinal cord. Importantly, FGF-receptor 1 is upregulated in spinal cord at a pre-symptomatic stage as well as in a mouse motoneuron-like cell-line NSC34 based model of SMA. Consistent with that, phosphorylations of FGFR-downstream targets Akt and ERK are increased. Moreover, ERK hyper-phosphorylation is functionally linked to FGFR-1 as revealed by receptor inhibition experiments. Our study shows that the FGF system is dysregulated at an early stage in SMA and may contribute to the SMA pathogenesis.

## Introduction

Juvenile Spinal Muscular Atrophy (SMA) is characterized by a loss of motoneurons in the ventral horn of the spinal cord, progressive muscle weakness and muscular atrophy. SMA is a monogenetic disease and all of the patients display deletions or mutations of the Survival of Motoneuron 1 *(Smn1)* gene [1], [2]. Humans possess one or more additional *Smn2* gene copies, which only differ in one translational silent mutation resulting in differentially spliced mRNAs [3], [4]. Only a small amount of functional full-length SMN protein derive from the *Smn2* gene, thereby only partially rescuing the SMA-phenotype [5]. Moreover, the number of *Smn2*-copies correlates positively with the amount of SMN-protein and the severity of the disease, which can be classified from severe (type I) to intermediate (type II) and mild (type III) [6].

With regard to the pathology of SMA, the most prominent feature of the disease is the degeneration of motoneurons. However, there is still an ongoing debate whether this is a motoneuron autonomous process or if there is a participation of other tissues. Interestingly, a muscle specific rescue of SMN does not lead to a significant benefit in survival and motor-function in a mouse model of SMA [7]. A motoneuron autonomous role of SMN is supported by experimental reduction of this protein in single motoneurons of zebrafish, which leads to axonal outgrowth and guidance defects [8]. However, some studies suggest a muscle intrinsic pathology as well as a contribution of the target muscle to motoneuron degeneration. In human SMA-fetus, a delay in muscle-development could be observed prior to morphological changes in motoneurons [9]. Moreover, a muscular SMN rescue in SMA-*Drosophila* models shows benefits regarding stability of neuromuscular junctions (NMJ) [10], [11]. Importantly, co-culture experiments demonstrate that myofibres derived from SMA-patients are less capable of preventing apoptosis of rat embryonic motoneurons than wild-type muscle-cells [12]. This study additionally suggests a supporting role of target muscle for motoneuron survival, most likely by secretion of neurotrophic factors.

An important system of such neurotrophic factors is the Fibroblast-Growth Factor (FGF) system. The FGF-system comprises four canonical FGF-receptor tyrosin kinases (FGFR) and 22 ligands. Besides their mitogenic effects, FGFs play important roles in embryonic development of several tissues, regulation of metabolic functions and control of homeostasis [13]. In muscle, FGFs control differentiation during development, regeneration in adult stages and carry out trophic functions [14], [15], [16]. With regard to neuronal systems, some FGFs are known to improve motoneuron survival either in a paracrine or autocrine fashion [17], [18]. In addition, they promote formation of neuromuscular junctions and axonal outgrowth [19], [20]. Widespread changes of FGF-family members have been previously demonstrated for pheochromocytoma cells (PC12) and as well as *in vivo* in forebrain and brain stem of neonatal rats after exposure to organophosphates [21], [22]. Thereby, the FGF-receptor tyrosin kinases act mainly through ERK- and Akt-pathways which promote neuronal survival as well as differentiation and neurite outgrowth (for review see [23]). The latter is mediated by Rho-kinase (ROCK)-pathway-signaling which in turn regulates actin-dynamics and stability of neuromuscular junctions [24]. Recently, we could show that the interaction of the SMN-protein with the neuronal actin-binding protein Profilin2a is important for proper ROCK-pathway-signaling and neurite outgrowth [25]. Importantly, ROCK-inhibition leads to prolonged survival in an intermediate SMA-mouse-model [26]. However, neuronal differentiation is not only controlled by extracellular FGF-ligands and transmembrane FGFR acting as classical membrane associated signal-complexes. FGFR-1 and its ligand FGF-2 can be imported into the nucleus where they regulate transcription, thereby controlling neuronal proliferation and differentiation [27]. Importantly, we could previously show an interaction of nuclear FGF-2 with SMN controlling the number of nuclear bodies (gems), which are known to be reduced in SMA-patients [28], [29], [30]. Moreover, in a *Drosophila* SMA-model, FGFR-orthologue *heartless* expressed in muscle has been indentified to act synergistically with SMN on motor endplate integrity [31].

Because of these evidences, we wanted to systematically elucidate the involvement of the FGF-system in SMA-pathogenesis. In the present study, we used quantitative reverse transcription PCR (qRT-PCR) to compare transcript levels of all 22 FGF-ligands and 5 receptors of severely affected SMA-mice with control animals during postnatal disease progression. Therefore, spinal cords and muscles as well as corresponding muscular and motoneuron-like cell line models of SMA were examined. In muscle, two of the main expressed FGFRs were downregulated. In spinal cord, all of the four canonical FGF-receptors were altered. Importantly, the highly expressed FGF-receptor 1 was not only upregulated in pre-symptomatic state at postnatal day 1, but also in a SMA cell-culture model of the motoneuron-like cell line NSC34. Consistent with that, an analysis of FGFR-downstream targets revealed hyper-phosphorylation of Akt and ERK. These results demonstrate widespread alterations of the FGF-system in muscle and spinal cord which could lead to a disturbed muscle-motoneuron communication. Further, these results show an involvement of the important ERK/Akt-pathways in SMA which do not only act on a posttranslational level – similar to the ROCK pathway – but also change gene-activity on a transcriptional level.

## Results

**Figure 1:**
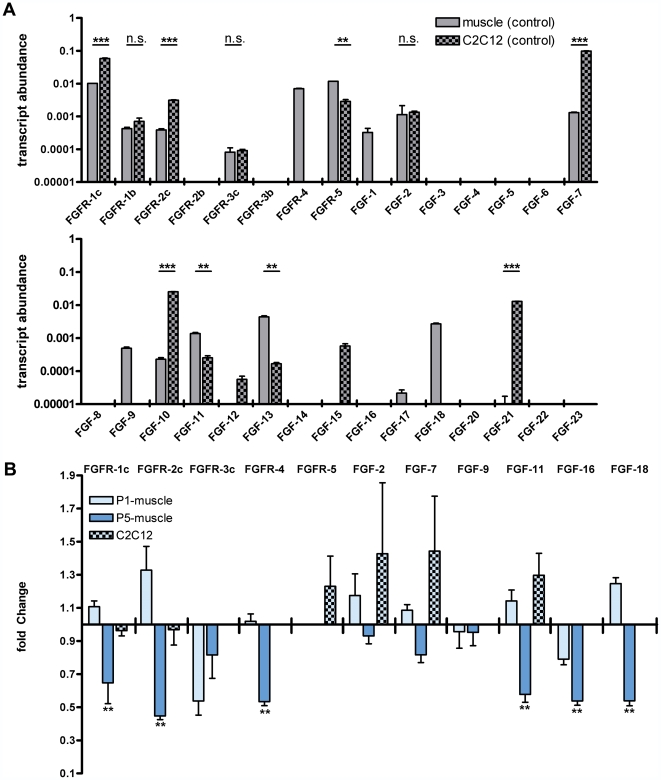
Expression and regulation of FGFs in muscle and C2C12-cells. (**A**) Transcript abundances relative to GAPDH in control muscle and scrambled siRNA transfected C2C12-cells. FGF-mRNA levels were measured by qRT-PCR in pooled samples of P5 control mice tissues and pooled samples of scrambled siRNA transfected cells relative to GAPDH as internal control. Transcript abundances were calculated from ΔC_T_-values. Unpaired t-tests of technical repetitions of measurements in cell culture compared to tissue abundances were performed (n = 2; n.s. = non significant, *p<0.05, **p<0.01, ***p<0.001). Bars represent means with standard deviations (SD). (**B**) Fold-change of FGF transcript levels in SMA-mice muscle and C2C12 cells after SMN-knockdown. FGF transcript concentration was measured by qRT-PCR in SMA-mice muscle and SMN-siRNA treated C2C12-cells. Transcript levels of SMA-mice and control animals were measured at P1 and P5. C2C12-cells were either transfected with SMN- or control scrambled-siRNA in 4 independent experiments (n = 4) with three replicates in each group. The knockdown for each experiment was monitored by western-blot analysis (Fig. S1). The fold-changes were calculated against each corresponding control group. Fold-changes of SMA-mice spinal cords were calculated against transcript levels of control mice of the same age. Results of SMN siRNA treated cells were compared to scrambled RNA transfected cells of the same experiment. SMA-mice transcript levels were tested against control animals by a Mann-Whitney test (n≥5, * p<0.05, ** p<0.01). mRNA-levels of SMN siRNA transfected cells were compared to scrambled siRNA transfected cells by a repeated measurements two way ANOVA (n = 4). Bars for fold changes represent means with standard error of mean (SEM).

**Figure 2:**
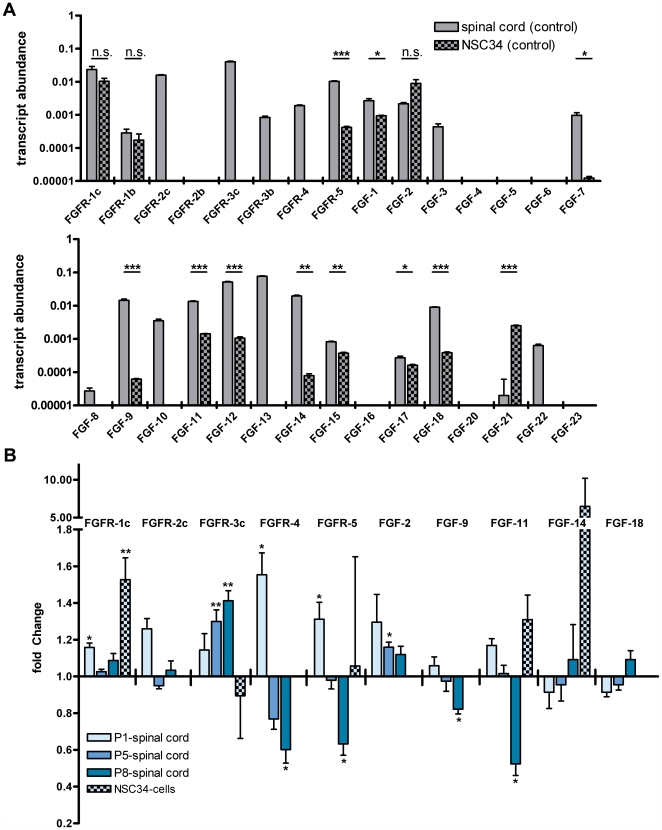
Expression and regulation of FGFs in spinal cord and NSC34-cells. (**A**) Transcript abundances relative to GAPDH in control mice spinal cords and scrambled siRNA transfected NSC34-cells. FGF mRNA levels were measured by qRT-PCR in pooled samples of P5 control mice tissues and scrambled siRNA transfected cells relative to GAPDH as internal control. Transcript abundances were calculated from ΔC_T_-values. Unpaired t-tests of technical repetitions of measurements in cell-culture compared to tissue abundances were performed (n = 2; n.s. = non significant, *p<0.05, **p<0.01, ***p<0.001). Bars represent means with standard deviations (SD). (**B**) Fold-change of FGF transcript levels in SMA-mice spinal cords and NSC34 cells after SMN-knockdown. FGF transcript concentration was measured by qRT-PCR in SMA-mice spinal cords and SMN-siRNA treated NSC34-cells. Transcript levels of SMA-mice and control animals were measured at P1, P5 and P8. NSC34-cells were either transfected with SMN- or control scrambled-siRNA in four independent experiments (n = 4) with three replicates in each group. The knockdown for each experiment was monitored by western-blot analysis (Fig. 3). The fold-changes were calculated in comparison to each corresponding control group. Fold-changes of SMA-mice spinal cords were calculated compared to transcript levels of control mice of the same age. Results of SMN siRNA treated cells were calculated compared to scrambled RNA transfected cells of the same experiment. SMA-mice transcript levels were tested against control animals by a Mann-Whitney test (n≥5, *p<0.05, **p<0.01). mRNA-levels of SMN siRNA transfected cells were tested against scrambled siRNA transfected cells by repeated measurements two way ANOVA (n = 4, **p = 0.0046). Bars for fold-changes represent means with standard error of mean (SEM).

**Figure 3:**
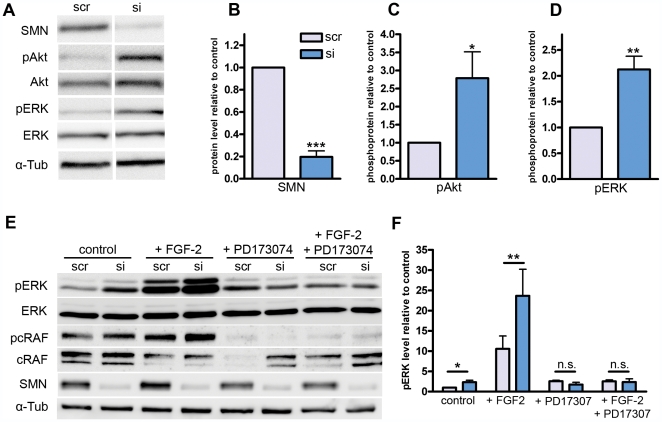
Western blot analysis of NSC34-cells after SMN-knockdown. (**A**) Phosphorylation of FGF-downstream targets Akt and ERK was analyzed in siRNA treated (si) and scrambled siRNA (scr) transfected cells. Phospho-antibodies against Akt (pAkt, S473), and ERK1/2 (pERK, T202,T204) were used to quantify changes in phosphorylation levels compared to non-phosphorylated Akt, ERK. Four independent experiments with three replications were performed. (**B**) Densitometrical measurements of SMN-bands normalized by α-tubulin showed an efficient knockdown of 20±5% in comparison to control-siRNA-transfected cells. (**C**) pAkt normalized to non-phosphorylated Akt was upregulated by a factor of 2.8±0.7. (**D**) pERK normalized to non-phospho-ERK was upregulated by a factor of 2.1±0.3. (**E**) The functional link between FGFR-1 signaling and ERK-hyperphosphorylation was analyzed by application of the specific FGFR-1 inhibitor PD173074 (50 µM). Additionally, FGF-2 was added to the medium in a final concentration of 50 ng/ml. (**F**) Densitometrical measurements of pERK normalized to non-phospho ERK revealed an upregulation of 10.6±3.1 fold in scrambled siRNA transfected cells under FGF-2 incubation compared to scrambled siRNA transfected control conditions. When transfected with SMN siRNA, the pERK level rises up to 23.7±0.6 fold change. These differences disappeared after PD173074 incubation. Bars and values represent means with standard error of mean (SEM). Significance was tested via repeated measurements two-way ANOVA (B, C, D, n = 4, ***p<0.0001, **p<0.01, *p<0.05) and paired ratio t-test (F, n = 6 for control conditions and n = 3 for remaining conditions, **p<0.01, *p<0.05).

To study alterations of FGF-system *in vivo* we employed SMA-mice modelling the genetic situation in SMA-patients. Homozygous SMN-depleted animals with a human transgene for *Smn2* (Smn^−/−^; SMN2^tg/+^) develop an SMA-type I like phenotype and die at a mean age of 10 days. Animals with a heterozygous murine depletion served as control animals (Smn^+/−^; SMN2^tg/+^) showing no obvious symptoms with a normal life expectancy [32], [33]. FGF-expression in spinal cords of SMA- and control-animals was examined at postnatal (P) days P1 (pre-symptomatic), P5 (symptomatic) and P8 (close to end stage), *quadriceps femoris* muscle at P1 and P5 via quantitative reverse transcription PCR (qRT-PCR) (Fig. 1, 2). However, as tissues represent a mixture of different cell-types, we employed cell line based models of SMA into our study by knocking down SMN by SMN-specific siRNAs. Cells transfected with scrambled control siRNA served as controls. The myoblastoma cell line C2C12 was included to model alterations of muscle-cells *in vitro* [34], while NSC34 cells were used as an *in vitro* model for motoneurons [35]. The knockdown efficiency for each biological repetition was determined by western-blot analysis with an anti-SMN-antibody for NSC34-cells (Fig. 3A, B) and C2C12-cells (Fig. S1). To determine differences between the *in-vivo* FGF-expression profile and the *in vitro* models, we compared transcript abundances relative to internal housekeeping gene glyceraldehyde 3-phosphate dehydrogenase (GAPDH) in spinal cord or muscle of control animals and respective cell lines (Fig. 1A, Fig. 2A). In a next step, we quantified fold changes of all FGF receptors and ligands in pooled samples. SMA-mice of each postnatal time point or each biological replication in case of cells lines were compared with their corresponding pooled control samples (data not shown). Transcripts which displayed alterations in one of these screening approaches were included in single sample studies by comparing transcript levels of single SMA-animals and biological replications of cell-line-models with respective controls (Fig. 1B, 2B).

### Members of the FGF family are down-regulated in SMA-mice muscle

To compare FGF expression profiles between C2C12 cells and muscle tissue, we determined transcript abundances relative to internal housekeeping GAPDH expression in pooled control samples of P5-mouse tissues and control-siRNA transfected C2C12 cells (Fig. 1A). In muscle 6 receptor-isoforms and 10 ligands were moderately to strongly expressed, while 4 of these genes (FGFR-4, FGF-9, FGF-17 and FGF-18) were not detected in C2C12-cells. On the opposite, FGF-12, FGF-15 and FGF-21 were much stronger or even exclusively expressed in C2C12-cells. Regarding those FGFs expressed in both, muscle and cells, FGFR-1b, FGFR-3c and FGF-2 displayed similar transcript abundances in muscle tissue compared to C2C12 cells. In contrast, FGFR-1c, 2c and ligands 7 and 10 showed a higher expression level within C2C12 cells whereas FGFR-5 and FGF-11 and 13 were more abundant in muscle.

Next, we analyzed 10 genes which were positive for alterations in the initial screening approaches of SMA-muscle against control muscle in single sample studies (Fig. 1B). While differences of pre-symptomatic P1 mice were not statistically significant (light blue bars, Fig. 1B) symptomatic P5 mice (dark blue bars, Fig. 1B) displayed significant downregulation of three receptors (FGFR-1c, FGFR-2c, FGFR-4) and three ligands (FGF-11, FGF-16, FGF-18). No significant differences of SMN knockdown and scrambled siRNA treated C2C12 cells were observed (checked bars, Fig. 1A). Interestingly, receptors 1c and 4, which were downregulated at P5 in SMA muscle, displayed relative high expression levels (Fig. 1A, B).

### FGF-dysregulations in SMA-mice spinal cords

A comparison of transcript abundances between control NSC34 cells and control mouse spinal cord revealed similar mRNA abundances for FGFR-1c, 1b and FGF-2 (Fig. 2A). In contrast, spinal cord expressed 7 receptor-isoforms and 16 ligands whereas only 3 receptor-isoforms and 11 ligands could be detected in NSC34 cells. Spinal cord expressed 4 receptor-isoforms and 5 ligands exclusively. In general, the spinal cord displayed a broader FGF and FGFR expression profile and higher overall expression levels compared to NSC34 cells, which might reflect the high cell type diversity within spinal cord tissue.

From 9 selected genes from the pooled cDNA screening, alterations of four receptors (FGFR-1c, FGFR-3c, FGFR-4, FGFR-5) and three FGF-ligands (FGF-2, FGF-9, FGF-11) were verified in individual SMA-animals at least at one postnatal time point (Fig. 2B). FGFR-4 and -5 as well as ligands FGF-9 and -11 displayed a similar pattern with regard to their developmental regulation. An upregulation at P1, which was significant for FGF receptors 4 and 5 and occured as a trend for FGF-9 and -11, was followed by a significant down-regulation at P8. In contrast, upregulation of FGFR-3c continuously increased during disease progression, whereby FGF-2 demonstrated a small, but significant change at P5. Importantly, upregulated FGFR-1c at pre-symptomatic stage P1 was also observed in NSC34 cells under SMN-knockdown conditions. Comparing *in vivo* and *in vitro* situations, these regulative effects were much stronger in NSC34-cells than in P1 spinal cords (Fig. 1B, light blue bars and checked bars).

### SMN-knockdown in NSC34 cells led to hyper-phosphorylation of FGFR-1 downstream targets Akt and ERK

Since we have found significant up-regulation of FGFR-1 both in SMA spinal cord and the SMA *in vitro* NSC34-model, we next addressed putative activation of FGFR-1 downstream targets. Therefore, we analyzed phosphorylation changes of Akt and ERK after SMN knockdown in NSC34 cells (Fig. 3A).

SMN-knockdown in NSC34 cells demonstrated its efficient down-regulation similar to levels observed in primary fibroblasts derived from type-1 SMA patients ([36], Fig. 3B). Consistent with the up-regulation of FGFR-1c transcript levels (Fig. 2B), we found Akt as well as ERK to be significantly hyper-phosphorylated (Fig. 3C, D), indicating over-activation of both pathways.

**Figure 4:**
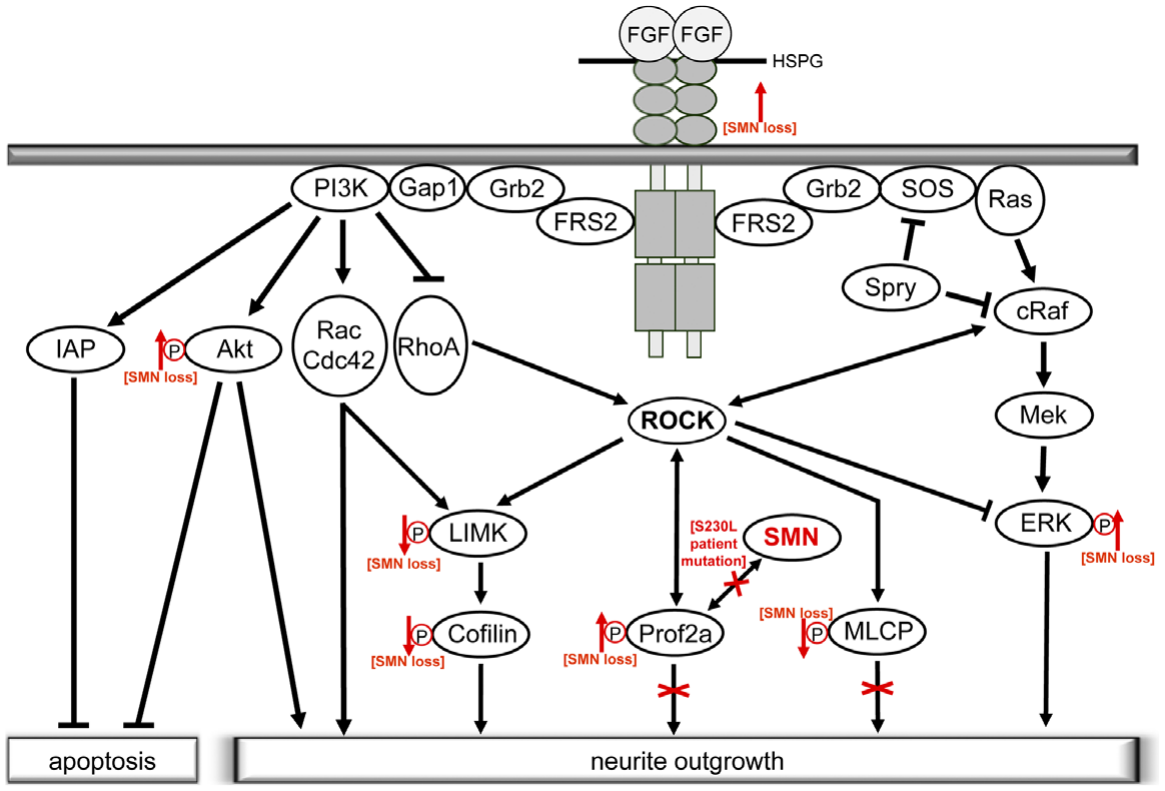
Crosstalk of neurotrophic factor signaling and ROCK pathway leading to neurite outgrowth – involvement of SMN protein. FGF-signaling promotes neurite outgrowth by small GTPase-dependent-, ERK- and Akt-pathways. Activation of the small GTPases Rac and Cdc42 and inhibition of the negative effector RhoA promotes outgrowth by posttranslational mechanisms. The ERK and Akt pathways, however, change transcriptional profiles towards an outgrowth promoting state. Both pathways functionally interact with RhoA-downstream-target Rho-kinase (ROCK) as a central node [58], [65]. ROCK thereby is important for integration and processing signals of the two major pathways involved in neurite outgrowth. Interestingly, recent findings of our group suggest a role of SMN-Profilin2a interaction in ROCK-pathway dependent outgrowth. SMN-reduction thereby leads to changes in phosphorylation-dependent regulation of ROCK-downstream targets indicated by red arrows [25]. Moreover, SMA-patient derived SMN-S230L-mutation does not interact with Prof2a and acts dominant negative on neurite outgrowth when overexpressed *in vitro* [25]. In this study, FGFR-1 was found to be upregulated under SMN-knockdown and consistently Akt and ERK showed hyper-phosphorylations. Thereby a sequestration of ROCK via Prof2a binding might enhance ERK-hyper-phosphorylation in a FGFR dependent manner.

In order to analyze the functional link between enhanced FGFR-1 expression and ERK-hyper-phosphorylation we applied the small compound PD173074 which binds the tyrosin kinase domain of FGFR-1 and efficiently inhibits its activity [37], [38]. As expected, addition of FGF-2 to the culture medium strongly increased pERK levels in both control and SMN-knockdown cells (Fig. 3E, F). Interestingly, the upregulation of pERK under SMN knockdown was maintained, supporting involvement of FGFR-1 upregulation in SMN-dependent ERK-hyperphosphorylation. Most importantly, this ERK-hyperphosphorylation disappeared under FGFR-1 inhibition by PD173074, both in FGF-treated and untreated cells which shows that FGFR-1 tyrosin kinase activity is responsible for accumulation of pERK. Efficient FGFR-1 inhibition was shown by hypo-phosphorylation of cRAF (Fig. 3E) which acts downstream of FGFR-1 (Fig. 4). These results show for the first time a SMN-knockdown induced upregulation of FGFR-1 expression which leads to an ERK-hyper-phosphorylation. Moreover, also pAkt accumulates which supports FGFR-1 upregulation being responsible for the observed effects. Furthermore, the involvement of these pathways in SMN-dependent processes such as neurite outgrowth and apoptosis points to a possible role of these central signal integrators in SMA-pathogenesis.

## Discussion

In this study, we could show multiple dysregulations within the FGF-system of a mouse model of SMA both in muscle and spinal cords. Importantly, the upregulation of FGFR-1c in spinal cord at presymtomatic state P1 could also be observed in an NSC34-cell culture model of SMA. Consistently, we could show a hyper-phosphorylation of FGFR-1 downstream targets ERK and Akt. The FGF-system comprises 22 ligands and 4 high affinity receptor tyrosin kinases [13]. A fifth receptor FGFR-5 has no intracellular kinase domain and might carry out regulatory functions by sequestering FGF-ligands from their high affinity receptor targets [39]. Importantly, our group could previously show an interaction of an intracellular FGF-2 isoform, FGF-2^23^ with the SMN-protein regulating stability of nuclear gems [28], [29], [30]. Nuclear FGF-2 is known to act on neurite outgrowth which is impaired in several SMA *in vitro* models possibly linking nuclear FGF to SMA pathology [40]. Regarding neuronal systems in general, the FGF-system is known to play important roles in neuronal survival, axonal guidance, target recognition and maturation of neuromuscular junctions (for review see [41]); processes which are affected by SMN. In muscle, FGFs carry out muscle intrinsic functions such as muscle cell survival as well as muscle derived support of motoneuron survival [42], [43], [44].

Our expression analysis of spinal cord and muscle tissues in comparison with corresponding motoneuron-like NSC34- and myoblastoma C2C12-cells, revealed considerable differences between *in vivo* and *in vitro* expression of FGF-ligands and receptors. As tissues represent a mixture of different cell types, it is most likely to detect FGF-expression of cell-types other than motoneurons or muscle cells. Thus, it is not surprising to find more FGFs to be expressed in tissues when compared with corresponding cell-lines. Only very few ligands are exclusively expressed in cell lines which might be attributable to the *in vitro* situation of cell culture or to their expression in blastoma cells. Because of normalization to GAPDH – which itself could be differentially expressed considering tissues and cell lines – this kind of analysis is intrinsically limited. However, since the observed differences for the FGF-system between expression in tissues and cell lines are relatively large, the data provide useful estimations for comparison of expression levels. Regarding expression in spinal cord, our results are in agreement with previous findings of our group, except for low expressed FGF-5 and 21 which were close to detection limit [45]. These observed differences might be due to genetic background effects of FVN or C57BL6 mice strain analyzed. The weaker elevation of FGFR-1c in P1 spinal cords of SMA-animals compared to NSC34-cells under SMN-knockdown might be a masking effect of non-regulated cell-types in tissue. As the upregulated FGFR-1 is the only FGF-receptor tyrosin kinase expressed in NSC34-cells, redundancy by other FGF-receptors can be excluded. Therefore NSC34-cells represent an excellent *in vitro* model for FGFR-1 functions.

In *quadriceps femoris* muscles, we could show a down-regulation of several receptors and ligands at P5. At this stage, it is unknown at which extent this particular muscle is affected by the disease complicating the integration of these events into the natural history of disease. However, our findings of downregulated receptors 1 and 4 might point to a role of FGFs in a muscle intrinsic pathology of SMA. An inhibition of FGFR-4 signaling in chicken embryos leads to impaired muscle cell progenitor differentiation and limb bud myogenesis [15]. FGFR-4 null mice, however, show no developmental phenotype [46], but more importantly an impairment of muscle regeneration mainly affecting myotube fusion [16]. FGFR-1 can only be found in differentiated postmitotic myoblasts of chicken embryos indicating a role in adult muscle [47]. Consistently, FGFR-1 mRNA is upregulated in less affected muscles during disuse-mediated skeletal muscle atrophy. *In vivo* overexpression of FGFR-1 protects from myofibre size decrease [14]. Interestingly, SMN-knockdown in C2C12 cells leads to reduced proliferation as well as defects in myoblast-fusion [48]. Moreover, SMA-fetus predicted to develop SMA-type I, show a delay in growth and maturation of myotubes prior to morphological changes occurring in spinal cords [9]. Thus, FGFR-1 and 4 downregulations in SMA-mice might fulfill negative modulating roles in myotube differentiation and muscular atrophy. However, although there is still an ongoing debate whether changes in muscle occur as a result of changes in motoneurons or denervation, there is growing evidence of a muscle intrinsic pathology in SMA. On a cellular level, the SMN-complex localizes to sarcomeric Z-discs both in *Drosophila* and mice. A role of the SMN-complex in Z-disc integrity, signaling to the nucleus and/or mRNP transport was suggested [49], [50]. Moreover, recent findings in a SMA mouse model comparing affected and unaffected muscles point to a muscle intrinsic pathology of SMA involving cellular survival pathways [51]. Interestingly, a muscular overexpression of insulin like growth factor 1 (IGF-1), which is known to lead to muscle-hypertrophy, also increases muscle mass in SMA-mice and enhanced survival rates [52], [53].

Several publications in recent years suggested an impaired muscle-dependent maturation and maintenance of the neuromuscular junctions (NMJ) during postnatal development in SMA. Muscle specific reduction of SMN resulted in lower numbers of synaptic boutons at the NMJ [10] and a muscular SMN-rescue improved survival of SMA *Drosophila* models [11]. *In vitro* experiments show that SMA-patient derived muscle cells are less capable of preventing apoptosis of rat primary motoneurons than controls [12]. These results indicate an impaired motoneuron-muscle communication caused by reduced SMN-levels in muscle cells leading to cell death in motoneurons. Remarkably, SMN-knockdown in *Drosophila* leads to reduced mRNA levels of the FGFR orthologue *heartless* (*htl*) in larval brains. Moreover, a mesoderm-specific (muscle) SMN-knockdown results in a reduction of postsynaptic accumulation of *htl* and a reduced number of synaptic boutons which could be rescued by mesoderm-specific overexpression of *htl* [31]. Importantly, the SMN-dependent reduction of *htl* in *Drosophila* is consistent with our findings of down-regulated FGFRs in SMA-mice muscle. However, as *Drosophila* only expresses two FGFRs and three ligand orthologues, these results are difficult to compare to mice. A possible role of postsynaptically expressed FGFRs in NMJ integrity of mice has not yet been investigated.

In spinal cord, we could show alterations of 4 receptors and 3 ligands. Importantly, the upregulation of FGFR-1 in spinal cord could be observed at the pre-symptomatic stage P1. NSC34 cells under SMN-knockdown resemble this regulation and show a sustained ERK1/2-activation. Expression of FGFR-1, which selectively signals through MAPK/ERK *in vitro*, is crucial for fiber outgrowth and guidance. FGFR-1 knockout motoneurons transplanted into neural tube of chicken embryos show severe guidance defects [54]. Importantly, motoneurons transfected with constitutively active MEK, an upstream activator of ERK, also showed defects in axonal guidance. Thus, proper guidance needs a fine regulation of FGFR-1 dependent signaling through the MEK/ERK-pathway [54]. Therefore, our observations of upregulated FGFR-1 in SMA-mice spinal cords might point towards SMN-dependent guidance and outgrowth defects. Interestingly, a zebrafish model of SMA with a SMN-reduction in single motoneurons leads to axonal outgrowth and guidance defects [8]. Moreover, we could recently show SMN-dependent changes in actin-dynamics and signaling pathways controlling neurite outgrowth [25], [55].

FGFR-1 is known to act via two pathways on neurite outgrowth, MEK/ERK and PI3K/Akt. Consistent with our findings of an FGFR-1 upregulation both, Akt and ERK were hyper-phosphosphorylated in NSC34 cells under SMN-knockdown. In PC12 cells, FGFR activation leads to a sustained ERK-activation and subsequently to neurite outgrowth [56]. PI3K/Akt and MEK/ERK pathways are both necessary for neurotrophic factor mediated axonal outgrowth [57]. Neurotrophic signaling, mediated by PI3K/Akt and MEK/ERK-pathways, finally activates transcription factors promoting neuronal differentiation as well as they directly signal to small GTPases Rac, Cdc42 and RhoA upstream of rho kinase (ROCK) (for review see [23]). In an intermediate SMA-mouse model, an inhibition of ROCK leads to improved NMJ-maturation and increased lifespan [26]. Moreover, our group could demonstrate changes in F-/G-actin ratios under SMN knockdown conditions in PC12 cells and motoneurons of SMA mice [25], [55]. Mechanistically, we could identify Profilin2a (Prof2a) as a binding partner of the SMN-protein. Since Prof2a also binds to ROCK, it links SMN-reduction with dysregulation of actin-dynamics (Fig. 4). Moreover, we could show widespread dysregulations within the signaling network regulating actin-dynamics leading to neurite outgrowth inhibition. Thereby, SMN reduction causes a release of Prof2a from SMN-Prof2a complex which in turn binds ROCK inducing a sequestration of ROCK from other downstream targets [25]. Interestingly, the ROCK pathway is also linked to the MEK/ERK-pathway (Fig. 4). In PC12 cells, an inhibition of ROCK leads to enhanced FGFR induced ERK-phosphorylation, which does not occur without any FGFR stimulus [58]. Similarly, a stimulus by ciliary neurotrophic factor (CNTF) and a simultaneous inhibition of ROCK results in hyper-phosphorylated ERK1/2 in retinal ganglion cells and in a Akt hyper-phosphorylation [59]. Thus, a sequestration of ROCK by enhanced Prof2a binding under SMN reduction and a simultaneous FGFR-1 upregulation explain the sustained ERK and Akt-phosphorylation observed in this study. While a transient ERK activation promotes neuronal survival, a sustained ERK activation might cause cell death suggesting a role of ERK in neurodegenerative processes (for review see [60]). Interestingly, our findings of upregulated FGFR-3c and FGF-2 also match a cell death promoting pattern. Both, FGFR-3 and FGF-2 knockout mice show less apoptosis of spinal ganglia sensory neurons after sciatic nerve axotomy implicating a negative modulating role of the FGFR-3/FGF-2 interaction on survival in neurodegenerative processes [61]. In accordance with that, apoptosis in retina cell development is induced by FGF-2. Moreover, FGF-9, which we could show to be downregulated in SMA-mice spinal cords, is known to be expressed in human and rat motoneurons [62] and *in vitro* experiments reveal a survival promoting role of FGF-9 on motoneurons [17].

Taken together, we could show widespread alterations within the FGF-system of SMA-mice muscle and spinal cords. Dysregulations in muscle might be associated with muscle-intrinsic functions such as myotube differentiation but also with NMJ-maintenance defects. Dysegulations in spinal cord might contribute to cell-death of motoneurons. Importantly, the upregulation of FGFR-1 could be modelled in NSC34-cells and most likely leads to hyperphosphorylation of FGFR downstream targets Akt and ERK. As both molecules are linked to ROCK-signaling and neurite outgrowth as well as they control cell death, they represent valuable targets of future investigations in the field of SMA.

## Materials and Methods

### Animals

The mouse mutant strain FVB.Cg-Tg(SMN2)2Hung SMN1*^tm1Hung^*/J [32] was purchased from the Jackson Laboratory (stock number 005058). To obtain 50% SMA-mice (Smn^−/−^;SMN2^tg/+^) and 50% control littermates (Smn^+/−^;SMN2^tg/+^), mice were bred and genotyped as described previously [25]. After decapitation, spinal cords and left and right *quadriceps femoris* muscles were dissected and immediately frozen in liquid nitrogen. All experimental protocols followed German law on animal care (study approval not necessary for tissue preparation as covered by § 4, Abs. 3 TierSchG).

### Cell Culture and transfection

Cells were incubated at 37°C in 5% humidified atmosphere. NSC34 cells were grown in Dulbecco's modified Eagle medium (DMEM) with low glucose content, 5% (v/v) fetal calf serum (FCS), 200 mM L-glutamine, 100 U/ml penicillin and 0.1 mg/ml streptomycin. For siRNA-knockdown, medium was changed to differentiation medium containing 1% (v/v) FCS instead of 5%. Immediately, siRNA-transfections were performed according to the manufacturer's recommendations using MetafectenePro (Biontex). C2C12-cells were maintained in high glucose DMEM containing 10% (v/v) FCS, 100 U/ml penicillin and 0.1 mg/ml streptomycin. For siRNA-transfections, medium was changed to differentiation medium containing 5% (v/v) horse serum instead of 10% FCS. Both NSC34 and C2C12-cells were harvested 72±2 h after siRNA-transfection. Three different SMN-siRNAs (synthesized by eurofins) were used for validation of results: siRNA2 (AUGCCUUUAGAAUAAAUAAA), siRNA3 (AAGAAGGAAAGUGCUCACAUA), siRNA4 (CAGAAGUAAAGCACACAGCAA) against murine SMN and scrambled control siRNA (GCGCAAAUAAACCGAAAGACA). No obvious differences in SMN-knockdown efficiency between different SMN siRNAs could be observed. FGFR-1 inhibitor PD173074 (Calbiochem, CAS 219580-11-7) was added 2 h prior to cell lysis in a final concentration of 50 µM. FGF-2 (PeproTech, Cat. 100-18-B) was added 10 min prior to lysis in a final concentration of 50 ng/ml. Cell-culture experiments depicted in figures 1, 2 and 3 A, B, C, D were repeated in four independent biological replications whereas three replications were carried out for experiments depicted in figures 3 E, F.

### RNA-Isolation and reverse transcription

RNA was isolated using the RNeasy Mini Kit (Qiagen) according to the manufacturer's recommendations. 2.5 µg of total RNA was reversely transcribed at 42°C in a total buffer-volume of 40 µl containing 3 µg random hexamer primers (Invitrogen), 200 U M-MLV-transcriptase (Invitrogen), 40 U RNase-Inhibitor (Agilent), 0.02 µmol dNTPs (Invitrogen) and 0.4 µmol DTT (Invitrogen). In a first step, RNA and random hexamer primers alone were incubated at 70°C followed by a rapid cooling step. Subsequently, the other components were added and incubated at 42°C for 1.5 h. Transcriptase was denatured by 15 min incubation at 70°C. For real-time PCR-applications, cDNA was diluted 1∶200.

### Realtime PCR

5 µl diluted cDNA, 7 µl of Power SYBRgreen (Applied Biosystems) and 2 µl primer dilution (1.75 µM each) were mixed in a 96-well MicroAmp reaction plate (Applied Biosystems). Realtime-PCR was performed using the StepOnePlus-thermocycler (Applied Biosystems). After an initial 10 min step of 95°C, PCR was performed for 40 cycles (15 s 95°C and 1 min 60°C). Primer sequences have been reported previously [45]. PCR-product specificity was verified by melt-curve analysis and compared to previous reported values [45]. Stability of three housekeeping genes was examined for each condition (Hprt1, Ppia, GAPDH). GAPDH-primers [63] were chosen for quantification of mRNA abundances in pooled samples of tissues and cell lines (Fig. 1A, Fig. 2A). Further, they were applied to quantify fold changes in tissues (Fig. 1B, Fig. 2B). Peptidylprolyl isomerase A (Ppia)-primers were chosen to quantify fold changes of cell lines (Fig. 1B, Fig. 2B, checked bars), because GAPDH displayed small but reproducible changes among the two conditions, whereas Hprt1 and Ppia were not regulated. C_T_-values were calculated with StepOne-software version 2.1 using a constant cycle threshold of 0.2. Quantification was performed using 2^−ΔΔC^_T_-method [64] giving fold changes in mRNA-levels relative to the arithmetic mean of the corresponding control group. Transcript abundances (Fig. 1A, Fig. 2B) relative to GAPDH were calculated by 2^−ΔC^_T_ for control conditions (P5 in case of animal tissues). Although a comparative interpretation of relative transcript levels between different transcripts is limited, it provides rough qualitative information about expression levels.

### Western blot

Cells were lysed with RIPA-buffer (137 mM NaCl, 20 mM Tris-HCl pH 7, 525 mM β-glycerophosphate, 2 mM EDTA, 1 mM sodium-orthovanadate, 1% (w/v) sodium-desoxycholate, 1% (v/v) Triton-X-100, protease inhibitor cocktail (Roche) and equal amounts of total protein (80–120 µg) in Laemmli-buffer (80 mM Tris-HCl pH 6.8, 2% SDS (w/v), 5% (v/v) 2-mercaptoethanol and 0.01% (v/v) bromphenol blue) were loaded on 10% polyacrylamide/SDS-gels. After electrophoretic separation, proteins were blotted on nitrocellulose membranes (GE Healthcare) and detected by horseradish-peroxidase linked secondary antibodies. Densitometric quantification of bands was performed with ImageJ software (version 1.43u). The following antibodies were used: mouse anti-SMN monoclonal (BD Biosciences), mouse anti tubulin (Santa Cruz Biotechnology, Inc.), rabbit anti pERK1/2 (T202, T204, Cell Signaling), mouse anti ERK1/2 (Cell Signaling), rabbit anti pAkt (S473, Cell Signaling), rabbit anti Akt (Cell Signaling), rabbit anti pcRAF (S338, Cell Signaling) and rabbit anti cRAF (Cell Signaling).

### Statistical analysis

Statistical analysis was done using GraphPad Prism 4 software. Mann-Whitney tests of fold changes of SMA-animals compared to control animals were performed for each postnatal time point individually. Realtime PCR of NSC34- and C2C12-cells was done in 4 independent biological replications with 3 cell culture repetitions for each group (SMN-siRNA-transfected and scrambled siRNA-transfected). Statistical significance was tested by repeated measures two-way ANOVA. Densitometric values of phosphoprotein-bands were normalized to those of the corresponding non-phosphorylated protein-bands. Four independent experiments with 3 repetitions of each group were quantified and tested by repeated measures two-way ANOVA for experiments depicted in figures 1, 2 and 3 A–D. At least three independent experiments were quantified and tested by paired ratio t-tests for experiments depicted in Fig. 3 E, F.

## Supporting Information

Figure S1 **SMN-knockdown in C2C12-cells.** (**A**) Anti SMN western-blots of SMN siRNA transfected C2C12 cells in comparison to scrambled siRNA-transfection. Four independent experiments with three replications were performed. (**B**) Densitometrical measurements of SMN-bands normalized to α-tubulin showed a knockdown of 37±2.9% in comparison to control-siRNA-transfected cells. Bars and values represent means with standard errors of mean (SEM). Significance was tested by repeated measurements two-way ANOVA (n = 4, ***p<0.001).
(TIF)
