# Supplementary figures and images for: Analysis of the Fibroblast Growth Factor System Reveals Alterations in a Mouse Model of Spinal Muscular Atrophy
Source: PLoS One. 2012 Feb 13;7(2):e31202. doi: 10.1371/journal.pone.0031202 (PMC3278439; doi:10.1371/journal.pone.0031202)

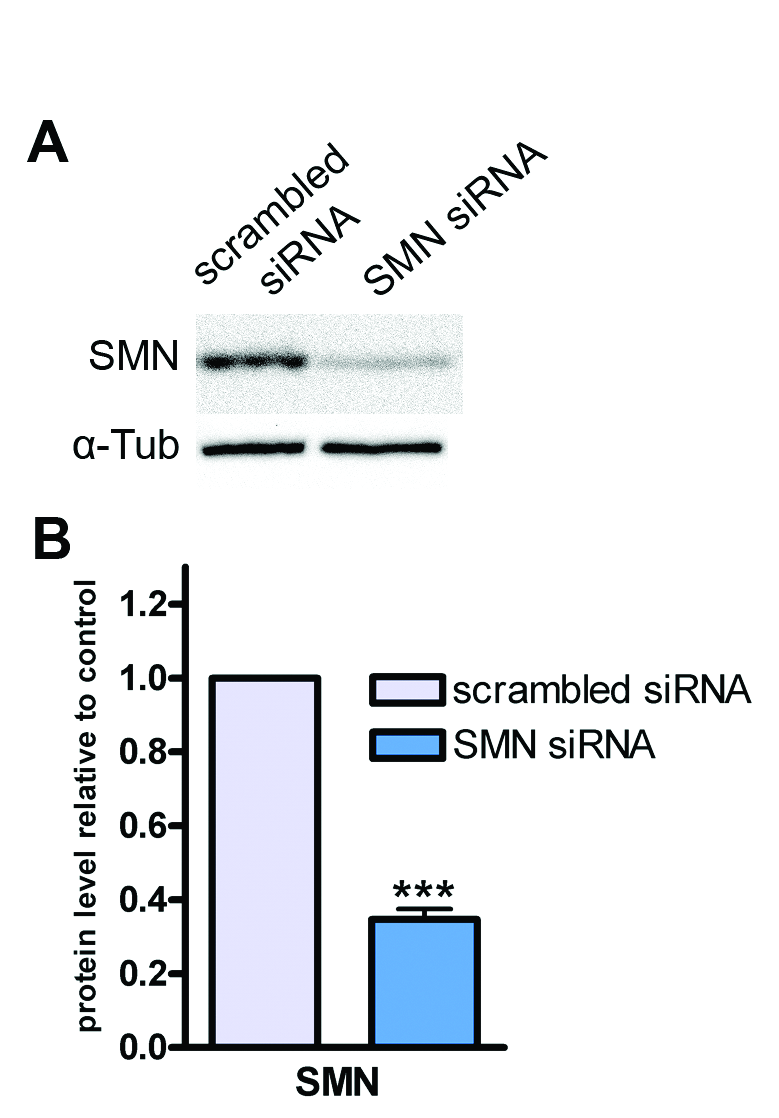

Supplement: Figure S1 — SMN-knockdown in C2C12-cells. (A) Anti SMN western-blots of SMN siRNA transfected C2C12 cells in comparison to scrambled siRNA-transfection. Four independent experiments with three replications were performed. (B) Densitometrical measurements of SMN-bands normalized to α-tubulin showed a knockdown of 37±2.9% in comparison to control-siRNA-transfected cells. Bars and values represent means with standard errors of mean (SEM). Significance was tested by repeated measurements two-way ANOVA (n = 4, ***p<0.001). (TIF) [file pone.0031202.s001.tif]
